# The point of normative models in judgment and decision making

**DOI:** 10.3389/fpsyg.2012.00577

**Published:** 2012-12-24

**Authors:** Jonathan Baron

**Affiliations:** Department of Psychology, University of PennsylvaniaPhiladelphia, PA, USA

In this comment, I shall try to summarize arguments that I have made before (Baron, 1985, 1994, 2004, 2006, 2008). These arguments are my attempt to state the standard view in the field of judgment and decision making (JDM).

JDM is applied psychology. The ultimate goal is to improve judgments and decisions, or keep them from getting worse. In order to achieve this goal we need to know what good judgments and decisions are. That is, we need criteria for evaluation, so that we can gather data on the goodness of judgments, find out what makes them better or worse, and test method for improving them when there is room for improvement. This is the main function of normative models.

Examples of normative models in JDM are:

1. For quantitative judgments (e.g., populations of cities, proportions of coin tosses that were heads): the normative model is simply the right answers. This also applies to relative judgments (which city has more people?) or judgments of category membership. We can also quantify departures from the right answers in various ways.
2. For judgments of the probability of unique events, one type of normative model, which is applied to a group of such judgments, scores the judgments by distance from 0 (no) or 1 (yes) and applies some formula to these scores. A related approach is to aggregate judgments with the same stated probability (e.g., all those with 80%), and ask if the proportion is correct (calibration, the proposition should be true 80% of the time).
3. Alternatively, for probabilities of related unique events, we can assess their coherence, their agreement with each other. If you say that the probability is 0.6 that X will win a competition and 0.7 that Y will win, you are not coherent.
4. For decisions, we can sometimes assess their consistency with basic principles of decision making, such as dominance (if A is better than B in some respects and worse in no respects, then choose A).
5. More typically, we assess the coherence of sets of decisions, using a mathematical model to define coherence, such as expected-utility theory or exponential discounting (for decisions over time). “Utility” is a summary measure of “good(ness).”

We could, in principle, define normative models in terms of the behavioral steps involved in making a good judgment or decision. For example, we could define the normative model for subtraction problems in terms of the steps of subtracting digits, regrouping, etc. But, as just illustrated, most normative models in JDM do not do this and are thus not computational, in the sense of being specified as procedures.

Note that some normative models concern coherence of responses with each other while others concern correspondence with the world, a distinction made first by Hammond (1996) [see Dunwoody (2009), for an overview]. Correspondence-type models are usually difficult to apply to decisions, so that are used mostly for judgments. This because the “right answer” to a decision question usually depends on the values of the decision maker.

JDM makes distinctions among three types of models: normative, descriptive, and prescriptive. The three-way distinction emerged clearly in the 1980s (Freeling, 1984; Baron, 1985; Bell et al., 1988—all of whom wrote independently of each other), although various parts of it were implicit in the writing of Herbert Simon and many philosophers (such as J. S. Mill).

Normative models, as noted, are standards for evaluation. They must be justified independently of observations of people's judgments and decisions, once we have observed enough to define what we are talking about. When not obvious, as in the case of simple correspondence (the “right answer”), they are typically justified by philosophical and mathematical argument (Baron, 2004). Particularly in cases where we want to quantify deviations from the single best response, several normative models may apply to the same case (e.g., scoring rules for probability judgments).

Descriptive models are psychological theories that try to explain how people make judgments and decisions, typically in the language of cognitive psychology, which includes such concepts as heuristics and strategies, as well as formal mathematical models. Within the three-model framework, descriptive models are most useful when they explain departures from normative models, so researchers often focus on the search for such explanations. Such models allow us to determine whether, and, if so, how, we might improve judgments and decisions. When a deviation from a normative model is found to be systematic, not just the result of random error, we call it a bias. For example, people are biased to choose default options, even when others are normatively equal or better.

Prescriptive models are designs for improvement. If normative models fall in the domain of philosophy (broadly defined) and descriptive models in the domain of empirical psychological science, then prescriptive models are in the domain of engineering (again, broadly defined). Originally, they were conceived as including mathematical tools that were useful for the formal analysis of decisions. These constitute the field of decision analysis, which includes several methods (and which has a society and a journal by that name). But prescriptive models can also be educational interventions (Larrick, 2004), which, for example, teach people alternative heuristics, to counteract heuristics that lead to biases.

A recent addition to the arsenal of prescriptive methods is the idea of “decision architecture” (Thaler and Sunstein, 2008), which consists of designing the presentation of decisions to those who will make them in such a way as to help people make the normatively better choice. A classic example is using the fact that people are biased toward the default to help them choose wisely by making what is usually the wise choice the default. For example, use a diversified portfolio as the default retirement plan for new employees (as opposed to, say, shares in company stock).

Thus, the ideal plan for JDM, sometimes actually realized (Baron, 2008; Thaler and Sunstein, 2008), is to apply normative models to judgments and decisions, looking for possible biases, then use the tools of psychology to understand the nature of those biases, and then, in the light of this understanding, develop approaches to improve matters. Of course, in real life these steps are not sequential, but are informed by each other. For example, decision analysis turns out to require the measurement of personal probability and utility, so now a large descriptive and normative enterprise is devoted to this measurement problem, which has produced better methods for measurement, which, in turn, are used to improve the original prescriptive models.

This plan clearly requires that the three elements are kept distinct. Suppose, for example, we make arguments for normative models on the basis of (descriptive) observations of what people do, under the assumption that people are rational. Then, we are likely to conclude that people are rational and that no prescriptive interventions are needed. The field of JDM would tend to disappear. Arguably, economics as a field made this assumption of rationality and thus was never concerned with helping people to make better economic choices, until recently, when economics has started to take the findings of JDM very seriously.

Another danger that JDM tries to avoid is to design prescriptive interventions without at least some clarity about normative and descriptive models. Specifically, we try to avoid “fixing things that ain't broke.” This sort of prescription has happened in psychology. For example, it was assumed that creativity was limited by a lack of divergent thinking (“thinking outside the box”), and many programs to improve creativity assumed this, despite the fact that the evidence indicate quite clearly that this was not a common problem [e.g., Johnson et al. (1968); and see Perkins (1981), for an overview].

Much of the debate within JDM is about the seriousness of various purported biases. Although strong advocates on one side or the other tend to think either that people are hopelessly biased or that we are perfectly adapted to our environment, more moderate folks think that, while it all depends on the person, the situation, and the task, there really are some situations where people can be helped, sometimes a lot, through the JDM approach (Thaler and Sunstein, 2008).

We need to keep normative and prescriptive models separate as well. If we assume that normative models are also prescriptive, they may become self-defeating. In decision making, the main normative standard is the maximization of (expected) utility, and the time required for calculation usually reduces utility. If normative models require elaborate calculation, then, when a real person attempts to apply one to a decision, the utility loss from the time spent may be greater than the gain from using the model, as opposed to some simpler heuristic. In many cases, then, normative models are applied by researchers, and real people may use various heuristics to improve their judgments as evaluated by the normative models (e.g., Davis-Stober et al., 2010).

On the other hand, summary versions of normative models may require no calculation at all and may serve the purpose of focusing attention on only what is relevant. For example, utilitarianism, a variant of utility theory that applies to decisions that affect many people, says that the goal of such decisions is to maximize total utility. A real person can often *save* time by simply asking, “Which option produces the best outcome on the whole, considering effects on everyone?” (Baron, 1990). Such a question is often easy to answer, and it can avoid more elaborate reasoning when, for example, this simple principle is must be weighed against another, non-utilitarian, principle such as “Do not use one person as a means to help another.” This conflict may occur in decisions about whether to abort a fetus, which would die anyway, in order to save the mother's life. When the fetal death is caused by abortion, then it is a means, and Catholic moral doctrine has been interpreted as prohibiting abortion for this reason, despite its obvious utilitarian benefit. The utilitarian solution is simpler because it involves only one principle and the decision maker does not need to resolve the conflict with another.
